# From Recognition to Production: Receptive and Expressive Cross-Situational Word Learning in Monolingual and Bilingual Children

**DOI:** 10.3390/bs16071080

**Published:** 2026-07-01

**Authors:** Kaityn Contino, Anna V. Duncan, Haley Weaver, Malvika Khandelwal, Alyssa Boucher, Kimberly Crespo

**Affiliations:** Sargent College of Health and Rehabilitation Sciences, Boston University, Boston, MA 02215, USA; annavd@bu.edu (A.V.D.); hjweaver@bu.edu (H.W.); malvi@bu.edu (M.K.); bouchera@bu.edu (A.B.)

**Keywords:** cross-situational word learning, bilingualism, statistical learning, speech production, input variability, children

## Abstract

This study examined how bilingualism and speaker variability influence children’s receptive and expressive cross-situational word learning (CSWL) performance. Effects of bilingualism were assessed both categorically (monolingual vs. bilingual) and continuously (length of bilingual experience). Ninety-five children aged 5–9 years completed a CSWL task under single-speaker and multiple-speaker conditions. Receptive performance was comparable between monolingual and bilingual children and was not influenced by speaker variability. However, within the bilingual sample, greater length of bilingual experience predicted stronger receptive learning. Expressive performance revealed a different pattern: monolinguals produced novel words more accurately than bilinguals. Within bilingual children, longer bilingual experience was associated with higher expressive accuracy. Speaker variability did not significantly affect expressive outcomes, nor did its effects interact with the language group or the length of bilingual experience. These findings suggest that children form representations during CSWL that support partial productions of newly learned words, while also indicating that expressive outcomes may be more sensitive to bilingual experience than receptive outcomes. Together, the results underscore the importance of considering both outcome modality and fine-grained measures of language experience when evaluating bilingual influences on novel word learning.

## 1. Introduction

To learn a new word, children must solve a two-part puzzle: identifying what the word refers to and learning how to say it. These components are related but not identical. Developmentally, children often demonstrate receptive knowledge, the ability to recognize and understand a word, before they are able to produce that word accurately (Bergelson & Swingley, 2015; Werker & Yeung, 2005). Although comprehension typically precedes production, the two develop in parallel (Benedict, 1979; Goldin-Meadow et al., 1976). Importantly, successful recognition of a word does not guarantee accurate production, as production places additional demands on phonological encoding, motor planning, and articulatory precision (Casserly & Pisoni, 2010; Levelt et al., 1999). For example, a child may reliably recognize a picture of a plate of spaghetti and correctly point to it in an array of pictures, demonstrating an intact receptive word–object link. However, when asked to name the same picture, the child may say “pasketti,” reflecting difficulty with phonological encoding and articulatory planning rather than a failure to recognize the word. Distinguishing between receptive and expressive word–object links is therefore critical for understanding children’s word-learning outcomes and for revealing how newly learned lexical representations are encoded and accessed.

Despite extensive research on word learning, most experimental studies have focused almost exclusively on receptive outcomes, including work examining learning via cross-situational word learning (CSWL; Yu & Smith, 2007). CSWL paradigms assess children’s ability to accumulate statistical regularities across ambiguous learning contexts to infer word–object mappings. In developmental (e.g., Fenson et al., 1994; Goldin-Meadow et al., 1976; Harris et al., 1995) and empirical (e.g., Gershkoff-Stowe & Hahn, 2013; Hu et al., 2025) studies, receptive performance often precedes or exceeds production. This gap is not generally attributed to a single factor, but rather to the additional demands that production places on lexical retrieval, phonological encoding, and motor planning (e.g., Heisler et al., 2010; Saletta et al., 2018; Vuolo & Goffman, 2018). While representations formed during CSWL may be sufficient to support receptive identification of referents (e.g., pointing or looking to the correct referent), successful production of target words likely requires the encoding of more phonologically detailed and specified mappings. Production performance may therefore provide a more stringent test of the quality of representations formed via CSWL. However, little is known about whether words learned through CSWL extend beyond recognition to support accurate production. Accordingly, the present study serves as both a replication and an extension of prior work: receptive CSWL was examined to replicate previous findings in a more representative sample of children, and this work was extended with the inclusion of an expressive outcome. By examining both receptive and expressive knowledge of novel words learned via CSWL, we provide a more complete account of how statistical word-learning mechanisms support the formation and use of novel lexical representations.

We were particularly interested in examining performance in bilingual children, who experience markedly different language-learning environments from their monolingual peers. Bilingual children are exposed to two languages, resulting in two separable but dynamically interactive phonological (e.g., Fabiano-Smith et al., 2021; Fabiano-Smith & Barlow, 2010; Keffala et al., 2018; Rodríguez-Guerra et al., 2023) and lexical–semantic (e.g., Floccia et al., 2020; Koutamanis et al., 2024; Rämä et al., 2024; Sheng et al., 2006) systems. This increased representational complexity may influence how new words are encoded, stored, and produced, particularly when learning occurs under ambiguity. To further probe the robustness of learned representations, we manipulated speaker variability because exposure to multiple speakers has been shown to strengthen phonological representations and support generalization and production (Apfelbaum & McMurray, 2011; Barcroft & Sommers, 2005; Höhle et al., 2020; Quam et al., 2017; Rost & McMurray, 2009). By comparing receptive and expressive word learning in monolingual and bilingual children, and by examining the role of speaker variability, this study examines how ambiguity, speaker variability, and differences in language experience shape children’s word-learning performance.

### 1.1. Cross-Situational Word Learning as a Window into Word Learning

CSWL captures how learners infer the meaning of words by tracking co-occurrences between words and their referents across multiple exposures without explicit instruction (Roembke & McMurray, 2016; L. Smith & Yu, 2008; Suanda et al., 2014; Yu & Smith, 2007; Zhang et al., 2021). CSWL paradigms are particularly informative because they approximate key features of naturalistic word learning: learners encounter words in varied contexts amongst several competitor referents rather than in perfectly structured pairings. This ambiguity allows researchers to assess how consistent patterns are extracted from noisy input.

In a typical CSWL experiment, participants are presented with multiple word–object pairings and gradually associate words with their referents by tracking co-occurrences across exposure trials. This paradigm has been validated across multiple methods, including computational modeling, observational data from parent–child interactions, and controlled laboratory studies across the lifespan (Blythe et al., 2010; Roembke & McMurray, 2016; K. Smith et al., 2011; L. Smith & Yu, 2008; Suanda et al., 2014; Vlach & Johnson, 2013; Yu & Smith, 2007; Zhang et al., 2021). Research has shown that both children and adults can reliably identify the correct referent for a word after repeated exposures, even when each trial includes multiple possible objects (Roembke & McMurray, 2016; L. Smith & Yu, 2008; Suanda et al., 2014). These findings demonstrate that CSWL supports robust receptive learning, but it remains unclear whether these representations are sufficiently specified to support production.

Research has shown that children and adults encode fine phonological detail during CSWL, and that this detailed encoding is associated with stronger performance. For example, Escudero et al. (2016) exposed adult learners to novel words that differed by a single phoneme (e.g., BON vs. TON), as well as novel words that differed by multiple phonemes. Participants learned correct word–referent pairs for all word types, demonstrating that adults encoded fine phonological detail during CSWL. In a follow-up study, Mulak et al. (2019) tested the relationship between CSWL task difficulty and phonological encoding by manipulating trial ambiguity within participants and found that learners retained sensitivity to subtle phonological differences between words across varying levels of trial ambiguity. Tuninetti et al. (2020) further extended this work by showing that adults correctly learned word–referent pairs that included both native and non-native phonological contrasts. While accuracy was above chance for all word types, accuracy was higher for perceptually easy than perceptually difficult items.

Taken together, these findings establish CSWL as a powerful mechanism for forming receptive word–object mappings, while also indicating that the precision of phonological encoding constrains the strength of newly formed word–referent associations. However, direct measures of production are largely absent, leaving a critical question unanswered: What phonological information do children encode during CSWL, and is it sufficient to support production of newly learned words? The present study addressed this gap by examining both receptive and expressive outcomes in children’s CSWL, with particular attention to how bilingual experience and speaker variability may shape performance.

### 1.2. Word Learning and Bilingualism

Bilingual children learn words within a fundamentally different linguistic environment than monolingual children. From infancy onward, bilingual children are exposed to two phonological and lexical systems that are distinct, but dynamically interacting (e.g., Fabiano-Smith et al., 2021; Fabiano-Smith & Barlow, 2010; Keffala et al., 2018; Rämä et al., 2024; Rodríguez-Guerra et al., 2023; Sheng et al., 2006). Empirical work has shown that bilingualism shapes how linguistic cues are weighted, and that differences in word-learning strategies emerge as early as infancy (Brojde et al., 2012; Byers-Heinlein & Werker, 2009; Houston-Price et al., 2010; Kalashnikova et al., 2018) and persist through early childhood (Kalashnikova et al., 2014). Despite this, across toddlerhood, preschool and the school-age years, many studies report comparable performance between bilingual and monolingual children on experimental novel word-learning tasks (Alt et al., 2013, 2019; Chan & Monaghan, 2025; Diego-Lázaro, 2021; Weatherhead et al., 2021). However, some findings indicate poorer performance in bilinguals compared to their monolingual peers (Gangopadhyay & Kaushanskaya, 2021). In other contexts, particularly in adulthood (e.g., Kaushanskaya & Marian, 2009b, 2009a) and occasionally in children (e.g., Singh, 2018; Singh et al., 2018), bilingual advantages in novel word learning are observed.

To date, few studies have examined the effects of bilingualism in CSWL (Crespo et al., 2023; Crespo & Kaushanskaya, 2021, 2025; Escudero et al., 2016; Li & Benitez, 2025; Neveu & Kaushanskaya, 2024), and findings have generally paralleled observations in the broader experimental word-learning literature. In most cases, monolinguals and bilinguals perform comparably on CSWL tasks. However, a small subset of studies report either poorer bilingual CSWL performance (e.g., Crespo & Kaushanskaya, 2021), or, conversely, bilingual advantages (e.g., Crespo et al., 2023; Crespo & Kaushanskaya, 2025; Escudero et al., 2016). The variability in outcomes across both CSWL and explicit word-learning paradigms likely reflect variations in task demands, stimulus characteristics, and learners’ language histories. Importantly, many of these studies have relied on receptive outcomes, most commonly picture identification tasks that index the strength of word–object mappings. Expressive outcomes have been examined less frequently than receptive outcomes; however, when assessed, findings suggest that production may be particularly sensitive to bilingual experience.

For example, work by Alt et al. (2013, 2019) has demonstrated that bilingual children may be more prone to production errors and accepting of phonological variation than monolingual peers. In Alt et al. (2019), in addition to other learning indices, monolingual and bilingual children were asked to produce the names of newly learned words and complete a mispronunciation detection task. Group differences were limited to production indices of learning; bilingual children were less accurate in producing novel words and showed greater tolerance of phonological variation (e.g., mispronunciations) in some contexts. However, in Alt et al. (2013), only preschool-aged bilingual children, but not school-aged bilingual children, were less accurate than monolingual peers in the naming task. School-aged bilingual children had nearly identical performance to monolingual peers. Taken, together, these findings suggest that bilingual experience may influence how phonological information is processed, represented, and accessed during word learning.

In the current study, children were classified as monolingual or bilingual, and within the bilingual group, we additionally quantified the length of bilingual experience (i.e., number of years a child has been exposed to both languages; (Crespo & Kaushanskaya, 2025; Meir & Armon-Lotem, 2013). Children with longer dual-language exposure may accrue greater experience coordinating and managing two lexical systems, (Byers-Heinlein & Werker, 2009; Kroll & Bialystok, 2013), which may engage and bolster cognitive processes that support both comprehension and production. Indeed, converging evidence indicates that bilingualism is not a uniform construct, and that variability in exposure, proficiency and language use predicts differences in both cognitive and linguistic outcomes (e.g., de Bruin, 2019; Yow & Li, 2015). At the lexical level, efficiency of access and retrieval is tied to language exposure and use (e.g., Schmidtke, 2014). By modeling bilingualism both categorically and continuously, we capture complementary sources of variance: group-based differences that reflect broad differences in language experience, and within-group gradients that index depth of exposure. This dual operationalization allows for a more precise characterization of how bilingual experience shapes CSWL that may otherwise be obscured by using only a group comparison approach.

Finally, speaker variability was a key manipulation in the current study because of its role in supporting word-learning performance (Apfelbaum & McMurray, 2011; Barcroft & Sommers, 2005; Crespo & Kaushanskaya, 2021; Höhle et al., 2020; Quam et al., 2017; Rost & McMurray, 2009). Exposure to multiple speakers can highlight consistent phonological patterns across voices, which may be particularly important when initially encoding new words (e.g., Rost & McMurray, 2009). By examining how speaker variability supports CSWL performance in both monolingual and bilingual children, we can gain insight into how characteristics of the input and language experience jointly shape the novel word learning across receptive and expressive contexts.

### 1.3. Word Learning and Speaker Variability

Relatively few studies have empirically tested the effects of speaker variability on CSWL performance. Across these studies, results generally suggest that both children and adults can accommodate speaker variability during learning with little impact to performance. Critically, as noted above, measures of word learning in these studies have been almost exclusively receptive, focusing on whether participants can select or identify the correct referent for each target word. It remains unknown whether speaker variability influences learning when the production of newly learned words is included as an outcome.

This distinction is important because evidence from phonetic training and early word-learning studies shows that exposure to multiple speakers can support word learning and generalization to new voices, indicating that speaker variability may help learners form more robust word-form representations (e.g., Barcroft & Sommers, 2005; Rost & McMurray, 2009). However, in the speech perception literature, studies have shown that listeners are slower and sometimes less accurate when processing words spoken by multiple speakers compared with a single speakers, suggesting that variability can impose additional cognitive and perceptual demands (Bradlow & Bent, 2008; Perrachione et al., 2011). Therefore, examining speaker variability in the context of expressive outcomes allowed us to determine whether its effects on CSWL differ as task demands shift from receptive to expressive contexts.

These dynamics may be further influenced by bilingual experience. Because bilingual children have more complex phonological and lexical systems, production may place different demands on the representations formed through CSWL. By comparing monolinguals to bilinguals, we tested whether receptive word–object representations were equally accessible for production across language groups, and whether speaker variability bolstered (or interfered with) performance similarly across groups.

### 1.4. The Current Study

The present study examined receptive and expressive performance in a CSWL task in English-speaking monolingual and Spanish–English bilingual children aged 5–9 years old. In addition to categorical group comparisons, we examined the length of bilingual exposure as a continuous predictor of both receptive and expressive outcomes. This individual-differences approach allowed us to go beyond simple group contrasts to test whether the amount of bilingual experience predicts performance in a graded and experience-dependent manner. In this study, children learned novel word–referent mappings in single-speaker and multiple-speaker conditions. Receptive performance was assessed using a two-alternative forced-choice referent selection task, and, in a subset of children, production was assessed via a picture-naming task. By assessing both receptive and expressive performance, this design allowed us to examine whether language experience and speaker variability differentially influenced these outcomes.

Our examination of receptive CSWL performance under single- and multiple-speaker conditions in monolingual and bilingual children is a replication of prior work; the receptive task used in the present study is similar to former paradigms (i.e., Crespo et al., 2023, 2024). Critically, the present sample consisted of a newly recruited, nationally sampled cohort of children, whereas our previous published work relied on samples that were primarily recruited from the Midwest (i.e., Wisconsin). This distinction is important because it improves the generalizability of the findings across a broader range of backgrounds and provides a stronger test of whether the observed effects are robust across heterogeneous experiences rather than specific to a single regional population. Based on previous research showing comparable performance between monolingual and bilingual children (e.g., Crespo et al., 2023) and adults (Li & Benitez, 2025; Neveu & Kaushanskaya, 2024), we expected similar performance across groups. Given the evidence from prior CSWL studies suggesting that multiple speakers neither facilitate nor interfere with learning (e.g., Crespo et al., 2023), we also expected children to demonstrate comparable receptive performance across single-speaker and multiple-speaker conditions.

To further explore individual differences, we examined the length of bilingual experience as a continuous predictor among bilingual children. Although previous studies have not found reliable group-level differences between monolingual and bilingual children in receptive CSWL performance, bilingual experience varies substantially within bilingual children. Because extended bilingual experience may provide greater practice learning across variable linguistic input, it is possible that the length of bilingual experience would be associated with stronger receptive learning even in the absence of group-level differences. We therefore hypothesized that greater bilingual exposure would be associated with stronger receptive performance. However, we did not have strong *a priori* hypotheses regarding how the length of bilingualism would interact with speaker variability to predict receptive performance.

Our second aim examined questions related to how language experience and speaker variability influenced the production of newly learned words. This aspect of the study represents a novel contribution, as prior work on CSWL has focused primarily on receptive outcomes. Accordingly, predictions regarding expressive performance were more exploratory and less clearly specified than those for receptive learning.

We considered that bilingual children experience distributed language exposure, resulting in phonological representations that are dynamically shaped through cross-linguistic interaction (e.g., Fabiano-Smith & Barlow, 2010; Fabiano-Smith & Goldstein, 2010), potentially influencing the encoding of newly learned words. Indeed, prior empirical work has shown that bilingual children have greater tolerance for phonological variation and produce newly learned words less accurately than their monolingual peers (e.g., Alt et al., 2019). Based on these findings, we hypothesized that bilingual children would show reduced accuracy in producing novel words relative to monolingual peers. In line with this theorizing, we anticipated that, within the bilingual sample, greater bilingual experience may yield poorer production of newly learned words. Because cross-linguistic influence arguably increases with more bilingual experience, we expected children with more bilingual experience to produce novel words less accurately than those with less bilingual experience.

Moreover, the current study extends prior work by directly examining whether speaker variability affects children’s production of newly learned words within the same paradigm used to assess receptive performance. This design allowed us to test whether expressive measures are more sensitive to input variability, even when receptive learning appears relatively robust. Given the evidence that exposure to multiple speakers may strengthen phonological and lexical representations and facilitate production (Apfelbaum & McMurray, 2011; Barcroft & Sommers, 2005; Crespo & Kaushanskaya, 2021; Höhle et al., 2020; Quam et al., 2017; Rost & McMurray, 2009), we hypothesized that children would more accurately produce words learned from multiple speakers than one speaker. Lastly, we did not have strong *a priori* hypotheses regarding whether the effects of speaker variability on production would differ across monolingual and bilingual children, or whether these effects would fluctuate across different levels of bilingual experience. Taken together, our analytical approach provided a more comprehensive test of how the language group, speaker variability, and individual differences in bilingual experience jointly shape children’s receptive and expressive word-learning outcomes.

## 2. Materials and Methods

### 2.1. Participants

This study was approved by the Charles River Campus Institutional Review Board at Boston University (6720E). Informed consent was obtained from participants’ legal guardians, and participants provided verbal assent. This study was conducted synchronously in online sessions via Zoom (Versions 5.14–5.17). Data and scripts for the current study are available on the Open Science Framework at https://osf.io/82n9q/overview?view_only=0837f53389e640de92ea1e48c299d1fe.

Ninety-five participants (52 female) aged 5–9 years old were recruited nationally. This age range was selected to ensure a range of bilingual experience. Inclusionary criteria included normal or corrected vision and normal hearing per parent report. Exclusionary criteria included a history of hearing loss, neurodevelopmental disorders, or neurological disorders.

Bilingual status was determined based on parent reports of children’s language exposure from a demographic questionnaire. Children were classified as bilingual if they had at least 20% exposure to Spanish or English. During data verification, four monolingual parents reported Spanish exposure; however, follow-up communication indicated that these estimates did not accurately reflect children’s language experiences and these children were thus classified as monolingual. Specifically, three caregivers reported more than 20% exposure to Spanish (Range: 21–50%). For these children, Spanish age of acquisition coincided with school entry and exposure estimates reflected time spent in school-based language classes. The fourth caregiver misunderstood the question, reporting exposure proportions that exceeded 100%. In all cases, it was confirmed with caregivers that children did not understand or speak Spanish with any fluency and were functionally monolingual. Because updated exposure estimates were not provided by the caregivers, the original reports that included Spanish exposure were retained for these four monolingual participants.

The final sample for receptive performance included 51 English monolingual (*M_Age_* = 7.70 years; 30 female) and 44 Spanish–English bilingual children (*M_Age_* = 7.74 years; 22 female). Fifty-four participants did not complete the production task. The CSWL task was part of a larger protocol that also included standardized language and cognitive assessments. Because task order was counterbalanced across participants, some children completed the CSWL task in the beginning of a session, whereas others completed the word-learning task at the end of a session. The production task was the last phase of the CSWL task. Consequently, some children refused to complete this phase when the task occurred last. As a result, the production analyses included 22 monolinguals (*M_Age_* = 7.50 years; 12 female) and 19 bilinguals (*M_Age_* = 7.50 years; 7 female).

Mothers’ years of education were used as a proxy for socio-economic status (SES) and were collected using the Language Experience and Proficiency Questionnaire (LEAP-Q, Marian et al., 2007). Children’s language history and current language exposure were collected through a demographic questionnaire. The Clinical Evaluation of Language Fundamentals—Fifth Edition (CELF-5; Wiig et al., 2013) was used to assess English language ability for both monolingual and bilingual children. Additionally, bilingual children completed the Clinical Evaluation of Language Fundamentals—Fourth Edition Spanish (CELF-4 Spanish; Wiig et al., 2006) to assess Spanish language ability. In the full sample, monolingual and bilingual children were matched on age, SES, nonverbal IQ, and overall language ability. Significant differences between the groups were observed in English-specific language ability, English age of acquisition and current English exposure. Participant characteristics for the full sample are reported in Table 1.

In the subset of participants included in the production analyses, monolingual and bilingual children were matched on age, overall language ability, English-specific language ability, and nonverbal IQ. Significant group differences were observed in SES, English age of acquisition, and current English exposure. Participant characteristics for the subset of participants included in the production analyses are reported in Table 2.

### 2.2. Length of Bilingual Experience

To calculate the length of bilingual experience, we subtracted the bilingual children’s age of acquisition in the second language from the child’s age at participation. For example, if a 7-year-old participant learned Spanish from birth and English starting at 3 years old, their length of bilingual experience would be 4 years. On average, our sample had 6.96 years of bilingual experience (*SD* = 1.67; range: 3.17–9.92).

### 2.3. Experimental Task

Participants completed a CSWL task that manipulated speaker variability within subjects. Stimuli presentation and data collection were conducted through Gorilla (www.gorilla.sc; Anwyl-Irvine et al., 2020) between May 2023 and March 2024. All novel words were English-like, as school-aged children in the United States typically show higher English exposure than Spanish exposure levels (Anderson, 2012; Castilla-Earls et al., 2019).

#### 2.3.1. Stimuli

Ten English-like nonwords were retrieved from Gupta et al. (2004), each following a CVCVC phonotactic structure; two lists of five nonwords were created. Novel words were matched on English and Spanish biphone probability and neighborhood density (calculated from the online CLEARPOND database) across lists. Audio stimuli for the nonwords were produced by 12 monolingual native English speakers (5 male, 7 female) ages 18–40 living in the Midwest of the United States who spoke Standard American English. Novel objects were selected from the Novel Object and Unusual Name (NOUN) Database 2nd Edition (Horst & Hout, 2016) and paired with each novel word. Word–object pair lists were counterbalanced across conditions. A full list of word–object pairings by condition can be found in the Supplemental Materials in Figure S1.

#### 2.3.2. Conditions

All participants completed two conditions: Single Speaker (SS) and Multiple Speaker (MS). In the SS condition, all novel words were produced by a single female monolingual English speaker. In the MS conditions, each novel word was spoken by 10 different speakers: 5 male and 5 female speakers. Speaker characteristics can be found in Table 3. Condition order and word–object pairs in each condition were counterbalanced. Each condition consisted of an exposure phase, receptive learning test phase, and, for a subset of the participants, an expressive test phase.

#### 2.3.3. Exposure Phase

An introduction video was presented at the beginning of the exposure phase that instructed children to look at, listen to, and learn the names of new toys. Children were exposed to 5 novel word–object pairs ten times across 25 trials in a pseudorandomized blocked order in each condition. Upon trial onset, two novel objects appeared right- and left-centered and the first novel word was produced (i.e., 0 ms). The second novel word was produced 2000 ms following trial onset, and each trial lasted approximately 6000 ms. Children received no information concerning which object was labeled by which word.

#### 2.3.4. Receptive Test Phase

Immediately following the exposure phase, children completed a test of receptive learning via a 2-alternative forced-choice task. Each word was tested twice across 10 trials, and each object functioned as a foil twice. Instructions were given once at the beginning of the test, informing children to select the image of the object that matched the word they heard. A 500 ms inter-stimulus interval preceded each trial. All target words within this test phase were produced by a single female speaker not heard in the exposure phase, and target words were produced once at 2000 ms following trial onset. The task progressed to the next trial after children selected a novel object or after 10,000 ms. Examples of exposure phase and receptive test phase trials are available in the Supplemental Materials in Figures S2 and S3.

#### 2.3.5. Expressive Test Phase

A test of expressive learning occurred immediately following the test of receptive learning. Children were shown one novel object at a time and asked to produce the target label. Each object was presented once in a pseudorandomized order through Microsoft PowerPoint.

### 2.4. Data Processing

Receptive test trials with reaction times (RTs) that were shorter than 150 ms and those that were longer than 10,000 ms were removed. Using this criteria, 90 trials were removed resulting in a total of 1810 usable trials.

For the expressive test trials, we adopted a phoneme-level analytical approach, consistent with prior developmental work (e.g., Pitt et al., 2024; Vuolo & Goffman, 2020). This approach allowed us to score segmentally variable and partially specified productions. Our rationale for using this method is that productions that deviate substantially from the target may still reflect underlying phonological knowledge when examined at the level of individual phonemes. Specifically, we calculated the number of correct phonemes produced (out of a possible five phonemes) by each participant for each word, such that participants received 1 point for every correct phoneme produced. Accuracy scoring was based on whether children’s productions matched the sequence of phonemes in target words, so for partial word productions, scoring began from the point at which the child’s production overlapped with the target. Diphthongs were counted as one vowel phoneme. Detailed examples of scored production trials can be found in Table 4. Trials in which a child did not respond were excluded from analyses. This resulted in the exclusion of 113 trials (27.56% of all expressive trials), yielding a total of 297 trials for the expressive task.

### 2.5. Analytic Approach

Receptive and expressive performance were modeled at the trial level. Receptive and expressive outcomes were analyzed separately in R (Version 2025.09.2+418) using the lme4 package. Two generalized linear mixed-effects models (GLMER) with a logit link function were fit to model the binary nature of the receptive data (e.g., correct [1] or incorrect [0] response) and the count-binomial nature of the expressive data (e.g., possible correct [1] or incorrect [0] production of each of the phonemes in a given word). While expressive performance was calculated as a continuous variable from 0 to 5, the underlying structure of the data reflects a binary distribution, such that participants have 5 independent opportunities to produce a correct or incorrect phoneme. This means that the model treats each phoneme as a success/failure trial and models the probability of success on a phoneme, not the total count directly. Therefore, model coefficients reflect changes in the log odds of correctly producing an individual phoneme within a target word. Accordingly, odds ratios greater than 1 indicate an increased likelihood of correct phoneme production, rather than the likelihood of producing one additional correct phoneme in the word.

In each model assessing group-level differences in performance, accuracy (i.e., receptive or expressive) was regressed on language group (contrast-coded; bilingual [−0.5] and monolingual [0.5]), speaker variability (contrasted coded; multiple speakers [−0.5] and single speaker [0.5]), and their interaction. Covariates, including age, mother’s education, English AOA, and English exposure were added sequentially and only retained if they explained significant variance as determined by model comparisons conducted using the anova() function in the stats package.

All models were fit with the maximal random effects structure and iteratively pruned using a model comparison approach (Muradoglu et al., 2023) until convergence was reached. The final model examining receptive performance included age and mother’s education, as well as by-subject and by-item random intercepts and a by-subject random slope for condition. The final model examining expressive performance included only age as a covariate and converged with by-subject and by-item random intercepts and random slopes for speaker variability.

Two additional GLMER models were constructed to investigate individual differences in performance for bilingual participants. Each model regressed accuracy (receptive or expressive) on length of bilingualism (mean centered), speaker variability (multiple speakers = −0.5 and single speaker = 0.5), and their interaction. The final model for receptive performance included by-subject and by-item random intercepts and a by-subject random slope for speaker variability. The model for expressive performance included by-subject and by-item random intercepts and slopes for speaker variability. The simplest models without any additional covariates were the best fitting models.

## 3. Results

Children learned words significantly above chance on the receptive task (*M* = 0.75, *SD* = 0.18, *t*(94) = 40.47, *p* < 0.01). Monolingual (*M* = 0.78, *SD* = 0.41) and bilingual (*M* = 0.73, *SD* = 0.44) children performed equally well (*t*(91.02) = −1.17, *p* = 0.25). Similarly, children were equally accurate (*t*(94) = −0.74, *p* = 0.46) in learning words after exposure to a single speaker (*M* = 0.76, *SD* = 0.22) and multiple speakers (*M* = 0.75, *SD* = 0.20).

Children produced significantly more correct phonemes than zero (*M* = 2.04, *SD* = 1.13, *t*(40) = 11.61, *p* < 0.01). On average, expressive performance did not differ by speaker variability condition (*t*(33) = 1.48, *p* = 0.15; single speaker: *M* = 1.97, *SD* = 1.22; multiple speakers: *M* = 2.32, *SD* = 1.54). However, monolinguals (*M* = 2.31, SD = 1.97) produced significantly more correct phonemes than bilinguals (*M* = 1.73, *SD* = 1.80; *t*(37.08) = −2.04, *p* = 0.05).

### 3.1. Receptive CSWL Performance

**Figure 1:**
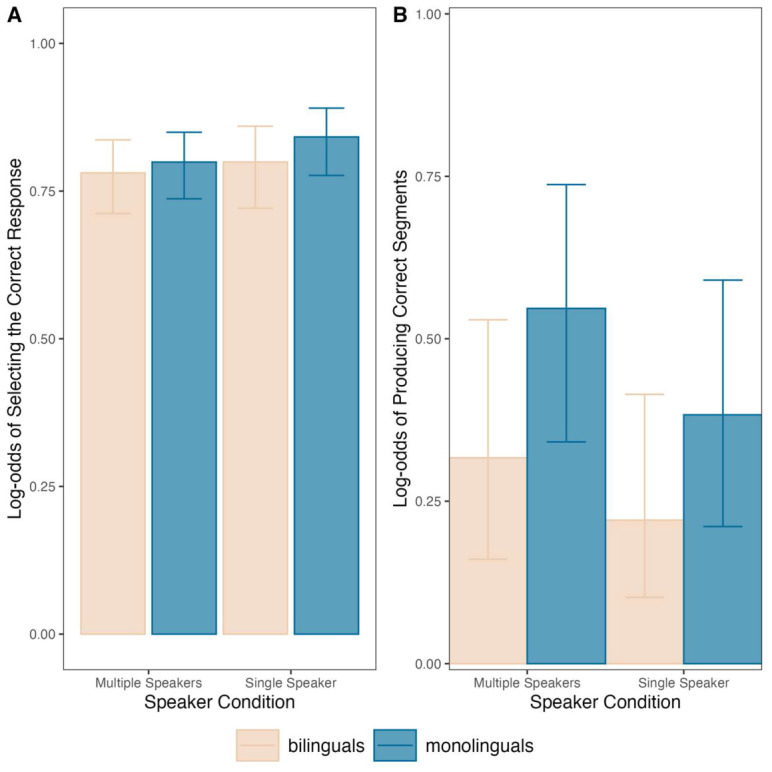
Receptive (**A**) and expressive (**B**) performance by speaker variability condition and language group. Graphs depict model-estimated effects, with error bars representing standard error.

Model results revealed that language group, speaker variability, and their interaction were not significant predictors of accuracy on the receptive task (*ps* > 0.05; Figure 1). Age and mother’s education, however, explained significant variance in performance (age: *b* = 0.41, SE = 0.07, *z* = 6.33, *p* < 0.05, odds ratio = 1.51, 95% CI [1.33, 1.72]; mother’s education: *b* = 0.09, SE = 0.03, *z* = 2.84, *p* < 0.05; odds ratio = 1.10; 95% CI [1.03, 1.17]). Older children and children whose mothers received more educational training had higher odds of identifying correct word–referent pairs on the receptive task. See Table 5 for model results for receptive performance.

**Figure 2:**
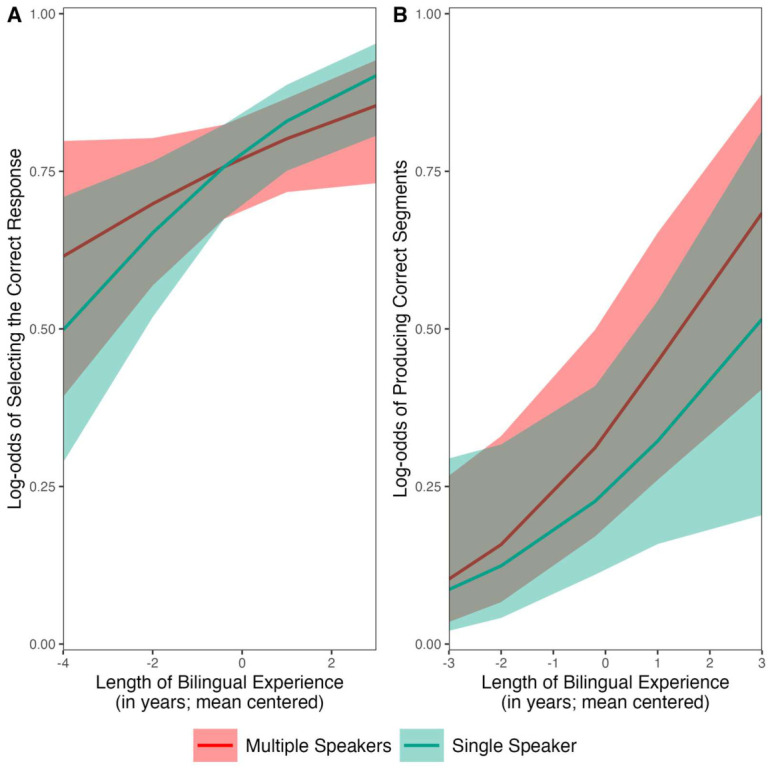
Receptive (**A**) and expressive (**B**) performance in the bilingual group only by speaker variability condition and length of bilingual experience. Graphs depict model-estimated effects, and error bars are plotted as +/−1 standard error.

In a separate model examining bilingual children only, results indicated a significant effect of length of bilingualism (*b* = 0.25, SE = 0.09, *z* = 2.77, *p* = 0.01, odds ratio = 1.29, 95% CI [1.08, 1.54]), suggesting that the odds of identifying correct word–referent pairs on the receptive task increased by 1.29 for every additional year of bilingual experience (Figure 2). The effect of speaker variability and the interaction term were not significant predictors of receptive performance (*ps* > 0.05). See Table 6 for model results for receptive performance within the bilingual group.

### 3.2. Expressive CSWL Performance

Model results revealed a significant effect of language group (*b* = 0.87; SE = 0.35, *z* = 2.49, *p* = 0.01, odds ratio = 2.39, 95% CI [1.20, 4.73]), such that the odds of producing more correct phonemes were two times higher for monolinguals than bilinguals (Figure 1B). Indeed, monolinguals produced an average of 2.31 correct phonemes (*SD* = 1.97), while bilinguals produced an average of 1.73 phonemes (*SD* = 1.80). There was also a significant effect of age (*b* = 0.45, SE = 0.12, *z* = 3.91, *p* < 0.01, odds ratio = 1.57, 95% CI [1.25, 1.96]), suggesting that the odds of producing correct phonemes were higher for older children than younger children. Neither the effect of speaker variability nor the interaction between speaker variability and language group reached significance. See Table 7 for model results for expressive performance.

In a separate model examining bilingual children only, results revealed a significant effect of the length of bilingualism (*b* = 0.45, SE = 0.14, *z* = 3.16, *p* < 0.01, odds ratio = 1.56 95% CI [1.18, 2.06]; *p* = 0.044; OR = 1.419), such that the odds of producing correct phonemes was 1.56 times higher for every additional year of bilingual experience (Figure 2B). No other effect reached significance. See Table 8 for model results for expressive performance within the bilingual group.

## 4. Discussion

The present study examined receptive and expressive CSWL performance in monolingual and bilingual children. The examination of receptive performance was a replication of our prior work with a more representative sample, while the inclusion of an expressive outcome was an extension. Investigating expressive CSWL represents a novel contribution, as prior research in CSWL has focused exclusively on receptive outcomes. Our approach examined bilingualism both categorically, comparing monolingual and bilingual children, and continuously, by modeling individual differences in the length of bilingual experience. Results indicated that receptive CSWL performance was comparable between monolinguals and bilinguals, and did not differ across single and multiple speaker conditions. However, within the bilingual sample, greater length of bilingual experience was associated with stronger receptive word-learning performance. Expressive outcomes suggested a divergence: monolinguals were more likely to accurately produce novel words than bilinguals. Nevertheless, within the bilingual group, longer length of bilingualism was associated with stronger expressive word-learning performance. Speaker variability did not influence receptive or expressive CSWL performance, nor did its effects differ across language groups or across varying levels of bilingual experience.

Taken together, these findings suggest that children encode phonological information during CSWL that support partial productions of newly learned words. Results further indicate that bilingualism does not constrain children’s ability to form word–referent mappings, but may influence the accessibility of newly learned lexical representations for production. Notably, cumulative bilingual experience, rather than categorical language group membership, appears to be a more sensitive predictor of variability in children’s CSWL performance.

### 4.1. Receptive CSWL

In the present study, receptive performance did not differ between monolingual and bilingual children, aligning with a large body of research demonstrating comparable performance between groups on both experimental novel word-learning tasks and CSWL tasks (Alt et al., 2013; Chan & Monaghan, 2025; Crespo et al., 2023; Weatherhead et al., 2021). Although categorical group comparisons revealed no differences between monolingual and bilingual children, analyses examining bilingual experience as a continuous predictor reveals a different pattern among bilingual participants, the length of bilingual experience was positively associated with receptive performance, such that children with more years of dual-language exposure had higher odds of selecting the correct referent at test.

One possibility is that increased experience managing two linguistic systems may strengthen mechanisms that support lexical learning, such as attentional control (Bialystok et al., 2012; Green & Abutalebi, 2013), sensitivity to distributional patterns (Antoniou et al., 2015; Kaushanskaya & Marian, 2009b), or efficiency in mapping phonological forms to referents (Bartolotti & Marian, 2017; Kaushanskaya & Marian, 2009b). It is also plausible that the observed effect of bilingual experience on CSWL may reflect greater English exposure (e.g., Bedore et al., 2016; Parra et al., 2011; Thordardottir, 2011). That is, children with longer histories of bilingual experience are also likely to have accumulated more exposure to English. This may have facilitated learning in the present task because novel words were phonologically similar to English. However, model comparisons revealed that English age of acquisition and English exposure did not improve model fits. English age of acquisition did not significantly predict production performance, and while English exposure did, the effect of the length of bilingualism remained significant. The consistent pattern of significance in both models suggested that the observed main effect of length of bilingualism on production performance was not reducible to earlier English age of acquisition or greater English exposure. Nevertheless, the results highlight the importance of considering bilingualism as a gradient construct rather than a categorical distinction. While the mechanisms underlying this effect remain unclear, modeling bilingual experience as a continuous variable allowed for more sensitive detection of variability in receptive learning outcomes.

Moreover, speaker variability did not significantly influence receptive performance in this nationally recruited sample of children. Children were equally likely to identify correct word–referent pairs learned from a single speaker and multiple speakers. This pattern is consistent with previous CSWL studies that included more regional samples showing that learners can accommodate multiple speaker input without impact to word-learning accuracy (Crespo et al., 2023; Crespo & Kaushanskaya, 2021). Given this trend in the literature, it is plausible that during CSWL, learners may prioritize extracting consistent associations between words and referents across trials while down-weighing variation in the phonetic realization of those words. Such cue weighing may allow learners to abstract phonological information from a variable acoustic signal. In this way, the statistical structure of the task may guide attention toward lexical cues, enabling children to form accurate receptive representations even in the presence of speaker variability.

Taken together, the findings for receptive performance indicate that children readily form word–object associations through CSWL regardless of monolingual or bilingual status or speaker variability input. At the same time, cumulative bilingual experience may strengthen CSWL, although the mechanisms underlying this effect remain unclear. This mixed pattern of findings underscores the importance of considering both categorical and experience-based measures and suggests that gradient conceptualizations of bilingualism may better capture meaningful variability in learning than group comparisons.

### 4.2. Expressive CSWL

A key question in the current study was whether children could produce words acquired via CSWL. Results revealed that children’s approximations of novel words included significantly more phonemes than zero, suggesting that children were engaging phonological encoding processes to support accurate word production. Although this pattern was not formally tested, descriptively, production performance appeared substantially lower than receptive performance overall, indicating that generating the phonological forms of newly learned words was more challenging than recognizing their referents. This dissociation between receptive and expressive performance aligns with theoretical accounts of word learning, suggesting that comprehension typically precedes production (Benedict, 1979; Goldin-Meadow et al., 1976). Successfully identifying the correct referent requires access to the lexical representation, whereas production places additional demands on phonological encoding, motor planning, and articulatory execution (Casserly & Pisoni, 2010; Levelt et al., 1999). As a result, representations that are sufficient for recognition may not yet be specified enough to support accurate production.

In contrast to the receptive CSWL findings, expressive performance differed across language groups. Monolingual children’s productions of newly learned words were significantly more accurate than bilingual children’s productions. This pattern is consistent with prior work showing that bilingual children tend to demonstrate lower production accuracy and greater tolerance for phonological variation in novel word-learning contexts (e.g., Alt et al., 2019). As discussed earlier, differences in English exposure may partially account for performance differences between monolinguals and bilinguals (Bedore et al., 2016; Parra et al., 2011; Thordardottir, 2011). Relative to monolingual children, bilingual children language exposure is distributed, which may reduce how efficiently they encode language-specific phonological details in any single language. Consequently, the English-like structure of the stimuli may have disproportionately impacted bilingual children’s ability to encode and retrieve word forms during the production task. Importantly, these findings should not be interpreted as evidence of weaker lexical learning among bilingual children; rather, they suggest that expressive retrieval of newly learned words may be particularly sensitive to differences in language experience.

Moreover, within the bilingual group, longer bilingual experience was associated with higher expressive accuracy, such that children with more years of dual-language exposure produced more correct phonemes. This finding suggests that cumulative bilingual experience may support the development of phonological and lexical processes that facilitate the production of newly learned words. Greater experience navigating two phonological systems may enhance children’s ability to encode and retrieve novel phonological forms, ultimately supporting more accurate production. It is important to acknowledge that our measure of bilingual experience is limited and may be confounded by participants’ age. Future research should explore alternative approaches to assessing individual differences in bilingual experience to better disentangle the effects of age and language exposure. Notably, this positive relationship emerged even though bilingual children, as a group, produced fewer phonemes than monolingual peers. Together, these findings highlight the importance of distinguishing between group-level differences and experience-based variation within bilingual populations.

As observed for receptive performance, speaker variability did not significantly influence expressive CSWL performance. Children produced novel words equally well regardless of whether the words were learned from a single speaker or multiple speakers. This result suggests that CSWL is robust against speaker variability across modalities; exposure to multiple speakers during learning neither substantially strengthens nor disrupts how children encode and retrieve phonological representations of newly learned words. This finding contrasts with previous research in speech perception and phonetic learning that has shown both facilitative and interference effects of speaker variability on learning (e.g., Apfelbaum & McMurray, 2011; Barcroft & Sommers, 2005; Bradlow & Bent, 2008; Höhle et al., 2020; Perrachione et al., 2011; Quam et al., 2017; Richtsmeier et al., 2009; Rost & McMurray, 2009). It is possible that the demands of the CSWL paradigm, particularly the need to track word–object associations under ambiguity, limited the extent to which variability in the acoustic signal influenced phonological encoding. Because children must resolve referential uncertainty, the cognitive load of managing multiple novel mappings may have overshadowed the effects of speaker variability on production. Future work is needed to clarify how acoustic variability interacts with the cognitive demands of resolving referential ambiguity, and to identify the conditions under which input variability facilitates or interferes with phonological encoding.

### 4.3. Limitations and Future Directions

As one of the first studies examining production outcomes in CSWL, and in bilingual children, future work with larger samples will be important for drawing stronger conclusions about how CSWL supports learning when production is the outcome, as well as how bilingual experience influences expressive learning. Another limitation worth noting is that the novel words used in the task were English-like. Future work should include stimuli reflecting both languages to better capture the full range of bilingual phonological experience and to determine whether word learning differs as a function of language-specific phonological structure or cross-linguistic overlap.

Moreover, cross-linguistic influences may lead bilingual children to produce phonemes that share some, but not all, phonetic features with the target (e.g., Fabiano-Smith & Goldstein, 2010). While our analyses focused on segmentally variable approximations of target words, we did not analyze phonemic error patterns (e.g., a child producing a consonant that differs in voicing compared to the target sound, but not in manner or place of articulation). Future analyses that also consider approximated phoneme productions, such as the method used by (Vuolo & Goffman, 2020), may reveal more subtle patterns in children’s production attempts and help clarify how cross-linguistic influences change with increasing bilingual experience.

Lastly, our continuous index of bilingual experience was derived from children’s chronological age. Because age predicted both receptive and expressive performance, the positive effects of bilingual experience on learning may partly reflect developmental effects rather than bilingual experience per se. In other words, a greater length of bilingualism may covary with general age-related improvements in attention, memory, speech production and other processes that also support word learning. Future research should test a narrower age range and use alternative indices, such as measures of proficiency, language exposure ratios, or patterns of language use to better isolate the effects of bilingual experience on learning outcomes. Incorporating such measures would allow for a more precise characterization of how different dimensions of bilingual experience contribute to variability in receptive and expressive CSWL performance.

## 5. Conclusions

In conclusion, the present study demonstrates that CSWL supports not only the formation of word–referent mappings that can be recognized receptively, but also the acquisition of phonological representations that support children’s approximations of newly learned words in production. Bilingual effects on word learning were more consistently captured by continuous measures of language experience rather than group membership, and these effects were most evident in expressive than receptive measures of learning. This pattern suggests that while children may form comparable receptive mappings during CSWL, differences may emerge more clearly when tasks require production of newly learned word forms. These findings highlight the value of examining production outcomes in addition to receptive outcomes in CSWL paradigms to more fully characterize the strength and usability of emerging lexical representations. More broadly, the results underscore the importance of considering both modality (receptive vs. expressive) and experience (continuous vs. categorical) when evaluating bilingual influences on word learning. Future work is needed to further investigate how bilingualism and input variability interact to shape the mechanisms underlying lexical acquisition.

## Figures and Tables

**Table 1:** Participant characteristics.

	Monolinguals		Bilinguals		
	*M* (*SD*)	*Range*	*M* (*SD*)	*Range*	*t*
*N*	51 (30 female)	-	44 (22 female)	-	-
Age	7.70 (1.51)	5.00–9.83	7.74 (1.56)	5.00–9.92	0.10
Caregiver Education (years)	20.00 (2.52)	14–24	17.39 (3.48)	9–25	−1.74
English AoA (months)	0.00 (0.00)	0–0	9.59 (14.29)	0–54	4.45 ***
Spanish AoA (months)	17.44 (25.45)	0–60	0.44 (2.57)	0–17	−2.00
Current English Exposure (%)	96.04 (9.79)	50–100	59.61 (13.90)	30–80	−14.41 ***
Nonverbal IQ ^a^	109.24 (15.37)	76–147	107.73 (13.67)	80–143	−0.51
English Language Ability ^b^	108.96 (15.66)	78–145	102.14 (15.47)	72–148	−2.13 *
Overall Language Ability ^c^	108.96 (15.66)	78–145	103.93 (15.98)	72–148	−1.54
	***n***		***n***		
Race					
*White/Caucasian*	37		29		
*Black/Afr. American*	5		2		
*Asian*	5		1		
*American Indian*	0		1		
*Pacific Islander*	0		1		
*Other*	4		10		
Ethnicity					
*Hispanic/Latino*	6		39		
*Not Hispanic/Latino*	45		5		
Dominance					
*English*	51		25		
*Spanish*	0		5		
*Equal*	0		14		

^a^ Visual Matrices subtest, Kauffman Brief Intelligence Test—2nd Edition. ^b^ Core Language Index standard score, CELF-5 English. ^c^ Highest Core Language Index standard score from either CELF-5 English or CELF-4 Spanish. In monolingual participants, this value is based on CELF-5 scores and is thus identical to values for English language ability. * *p* < 0.05; *** *p* < 0.001.

**Table 2:** Production subset participant characteristics.

	Monolinguals		Bilinguals		
	*M* (*SD*)	*Range*	*M* (*SD*)	*Range*	*t*
*N*	22 (12 female)	-	19 (7 female)	-	-
Age	7.50 (1.47)	5.00–9.75	7.67 (1.63)	5.00–9.92	0.34
Caregiver Education (years)	19.91 (2.67)	16–24	17.39 (4.13)	9–25	−2.27 *
English AoA (months)	0.00 (0.00)	0–0	9.58 (15.64)	0–54	2.67 *
Spanish AoA (months)	42.00 (25.46)	24–60	0.08 (0.34)	0–1.5	−2.33
Current English Exposure (%)	98.32 (4.86)	79–100	59.21 (15.92)	30–80	−10.30 ***
Nonverbal IQ ^a^	111.23 (16.03)	76–147	107.26 (15.55)	80–143	−0.80
English Language Ability ^b^	113.41 (15.79)	79–145	106.37 (18.86)	72–148	−1.28
Overall Language Ability ^c^	113.41 (15.79)	79–145	107.63 (18.95)	72–148	−1.05
	***n***		***n***		
Race					
*White/Caucasian*	16		16		
*Black/Afr. American*	1		0		
*Asian*	3		0		
*Other*	2		3		
Ethnicity					
*Hispanic*	2		16		
*Not Hispanic*	20		3		
Language Dominance					
*English*	22		12		
*Spanish*	0		0		
*Equal*	0		7		

^a^ Visual Matrices subtest, Kauffman Brief Intelligence Test—2nd Edition. ^b^ Core Language Index standard score, CELF-5 English. ^c^ Highest Core Language Index standard score from either CELF-5 English or CELF-4 Spanish. In monolingual participants, this value is based on CELF-5 scores and is thus identical to values for English language ability. * *p* < 0.05; *** *p* < 0.001.

**Table 3:** Speaker characteristics.

Speaker	Fundamental Frequency (F0, Hz)	Min Pitch (Hz)	Max Pitch (Hz)	Word Duration (s)
***Single Speaker***				
Female Speaker 1	256.90	158.66	278.09	0.97
Female Speaker 2	218.70	166.61	271.62	1.07
***Multiple Speaker***				
Female Speaker 3	232.50	171.22	251.11	0.98
Female Speaker 4	223.80	151.12	297.63	1.15
Female Speaker 5	238.80	185.66	258.79	1.06
Female Speaker 6	239.10	189.38	252.53	1.16
Female Speaker 7	224.60	178.29	246.88	0.84
Female Speaker 8	251.88	169.25	290.05	1.17
Female Speaker 9	212.11	167.57	273.99	0.97
Female Speaker 10	208.42	156.46	212.83	1.04
Female Speaker 11	245.72	169.88	267.42	0.93
Female Speaker 12	211.88	167.50	228.39	1.10
*M_Females_*	*228.88*	*170.63*	*257.96*	*1.04*
Male Speaker 1	123.77	117.36	130.39	1.00
Male Speaker 2	114.44	109.96	119.79	0.87
Male Speaker 3	121.09	85.86	126.09	1.03
Male Speaker 4	123.87	87.43	142.35	0.97
Male Speaker 5	110.98	92.61	124.80	0.72
Male Speaker 6	145.62	85.65	158.04	0.90
Male Speaker 7	123.68	103.18	145.30	1.01
Male Speaker 8	106.81	78.60	113.31	0.91
Male Speaker 9	128.94	114.61	132.47	0.85
Male Speaker 10	115.72	102.77	127.64	0.93
*M_Males_*	*121.49*	*97.80*	*132.02*	*0.92*
***Testing Speaker***				
Female Speaker 13	222.40	183.49	233.16	0.89

**Table 4:** Production trial scoring examples.

Scoring Example	Target	Production	Score
Correct target	/bæsɪm/	/bæsɪm/	5
Target with incorrect vowel	/taɪnʌf/	/tinʌf/	4
Target with incorrect consonant	/kimɪg/	/timɪg/	4
Target with multiple incorrect phonemes	/gonip/	/gɪtik/	2
Partial target (first syllable)	/baɪlɑb/	/baɪl/	3
Partial target (second syllable)	/keɪdæd/	/dæk/	2

**Table 5:** Model results for receptive performance.

Variable	Estimate	SE	*z* Value	*p* Value
Intercept	1.43	0.14	10.18	<0.01
Age	0.41	0.07	6.33	<0.01
Mother’s Education	0.09	0.03	2.84	<0.01
Speaker Variability	0.20	0.15	1.34	0.18
Language Group	0.20	0.20	0.98	0.33
Speaker Variability: Language Group	0.18	0.28	0.63	0.53

**Table 6:** Model results for receptive performance, bilinguals only.

Variable	Estimate	SE	z Value	*p* Value
Intercept	1.24	0.18	6.80	<0.01
Speaker Variability	0.05	0.20	0.27	0.79
Length of Bilingualism	0.25	0.09	2.77	0.01
Speaker Variability: Length of Bilingualism	0.13	0.11	1.16	0.25

**Table 7:** Model results for expressive performance.

Variable	Estimate	SE	*z* Value	*p* Value
Intercept	−0.65	0.32	−2.01	0.04
Speaker Variability	−0.58	0.41	−1.41	0.16
Language Group	0.87	0.35	2.49	0.01
Age	0.45	0.12	3.91	<0.01
Speaker Variability: Language Group	−0.17	0.60	−0.29	0.78

**Table 8:** Model results for expressive performance, bilinguals only.

Variable	Estimate	SE	*z* Value	*p* Value
Intercept	−0.92	0.37	−2.52	0.01
Speaker Variability	−0.45	0.41	−1.10	0.27
Length of Bilingualism	0.45	0.14	3.16	<0.01
Speaker Variability: Length of Bilingualism	−0.09	0.20	−0.42	0.67

## Data Availability

Data and scripts for the current study are available on the Open Science Framework at https://osf.io/82n9q/overview?view_only=0837f53389e640de92ea1e48c299d1fe.

## Supplementary Materials

The following supporting information can be downloaded at https://www.mdpi.com/article/10.3390/bs16071080/s1, Figure S1: Word-object pairs by order; Figure S2: Example exposure and test trials by condition for order A; Figure S3: Methodological details for exposure and test trials.

## Abbreviations

The following abbreviations are used in this manuscript:

CSWL	Cross-Situational Word Learning
CELF-5	Clinical Evaluation of Language Fundamentals—Fifth Edition
KBIT-2	Kauffman Brief Intelligence Test—2nd Edition
