# Supplementary material for: From Recognition to Production: Receptive and Expressive Cross-Situational Word Learning in Monolingual and Bilingual Children
Source: Behav Sci (Basel). 2026 Jul 1;16(7):1080. doi: 10.3390/bs16071080 (PMC13405757; doi:10.3390/bs16071080)
Supplement: Supplementary file 1 [file behavsci-16-01080-s001.zip › Supplementary S1.pdf]

Figure S1

Word-object pairs by order

List A		List B	
Gonepe	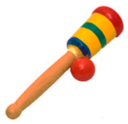	Gabek	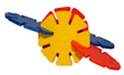
Basim	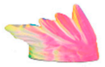	Bilob	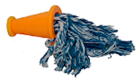
Kemig	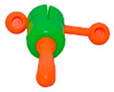	Kadad	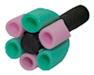
Dofege	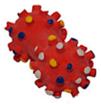	Denose	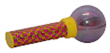
Tinuf	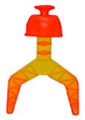	Tafat	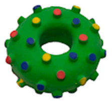
