# Supplementary material for: From Recognition to Production: Receptive and Expressive Cross-Situational Word Learning in Monolingual and Bilingual Children
Source: Behav Sci (Basel). 2026 Jul 1;16(7):1080. doi: 10.3390/bs16071080 (PMC13405757; doi:10.3390/bs16071080)
Supplement: Supplementary file 1 [file behavsci-16-01080-s001.zip › Supplementary S2.pdf]

Figure S2

Example exposure and test trials by condition for order A

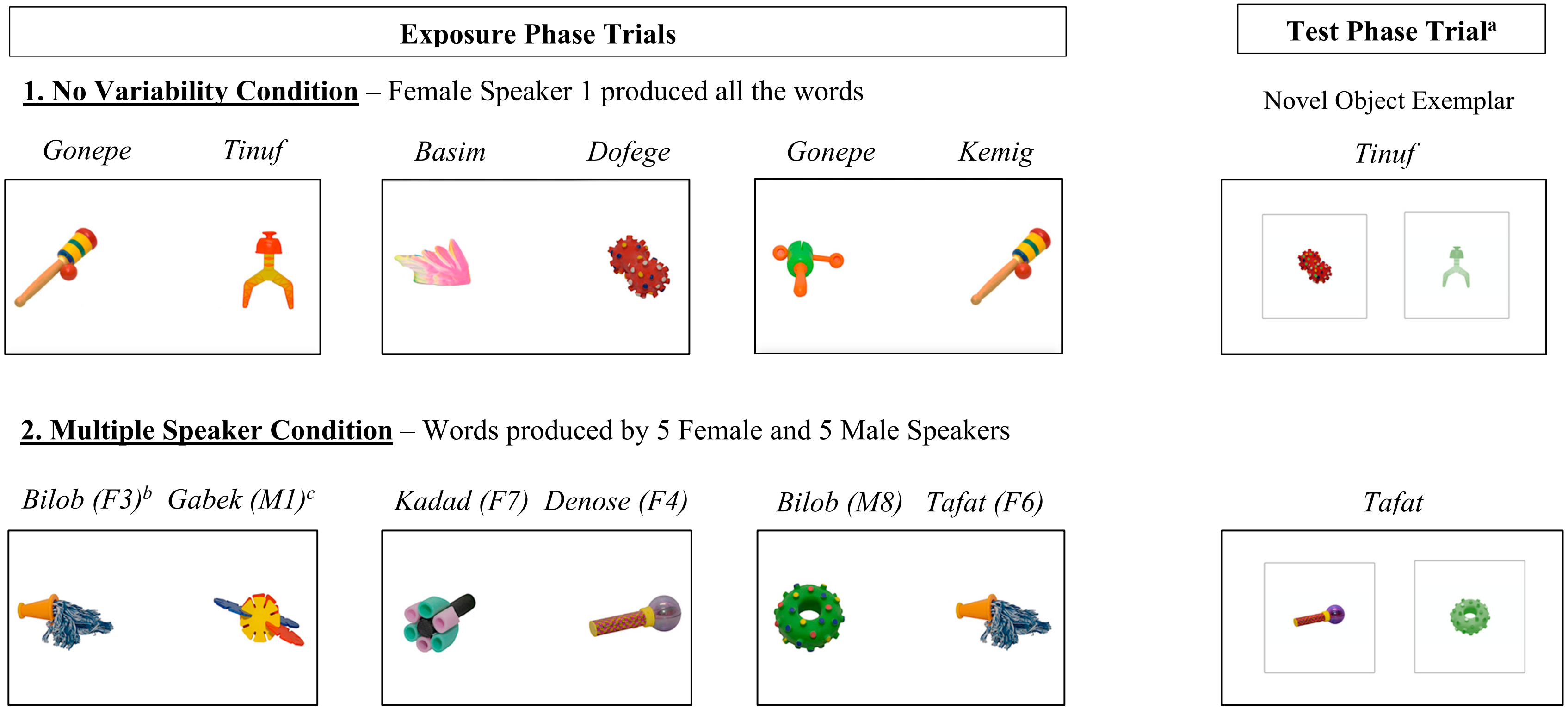

Note. This graphic depicts three exposure trials and one test trial for each condition for Order A. Word Lists and Picture Lists were counterbalanced to create other permutations of each condition for Order B. The first word produced in each trial did not always label the left-centered object first. In these instances, the first word produced labeled the right-centered object and the second word produced labeled the left-centered object.

[a] Female Speaker 13 produced all test words

[b] (F3) = Female Speaker 3

[c] (M1) = Male Speaker 1
