# Supplementary material for: From Recognition to Production: Receptive and Expressive Cross-Situational Word Learning in Monolingual and Bilingual Children
Source: Behav Sci (Basel). 2026 Jul 1;16(7):1080. doi: 10.3390/bs16071080 (PMC13405757; doi:10.3390/bs16071080)
Supplement: Supplementary file 1 [file behavsci-16-01080-s001.zip › Supplementary S3.pdf]

Figure S3

Methodological details for exposure and test trials

C1. Exposure Phase

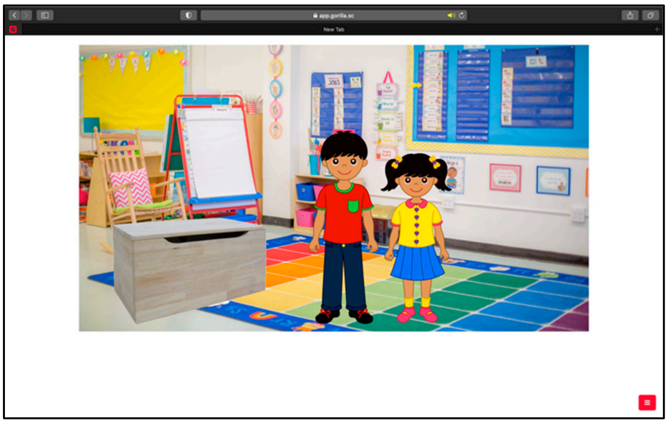

Instructions: *"Hi! Let's play a game! In this game, you'll see some new toys. To win this game, you'll have to learn the names of these new toys! Let's look!"*

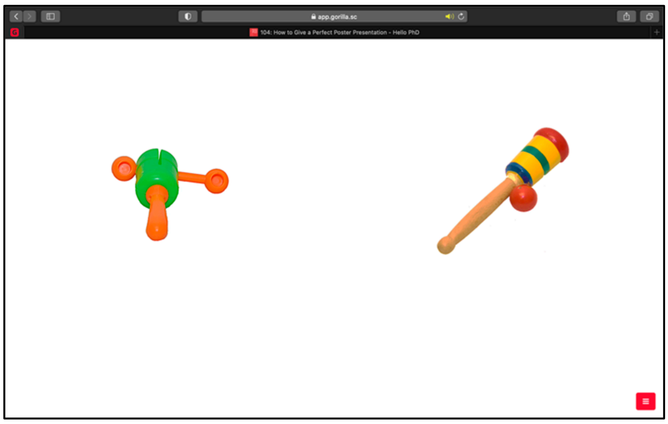

"Gonepe" (0 ms)

"Kemig" (2000 ms)

Time in Trial (6000ms)

Note. Instructions were only presented once at the beginning of the task. After, in each exposure trial, two objects were presented, and the first novel word was produced at trial onset (i.e., 0 ms). The second novel word was produced 2000 ms after trial onset. Each exposure trial was approximately 6000 ms.

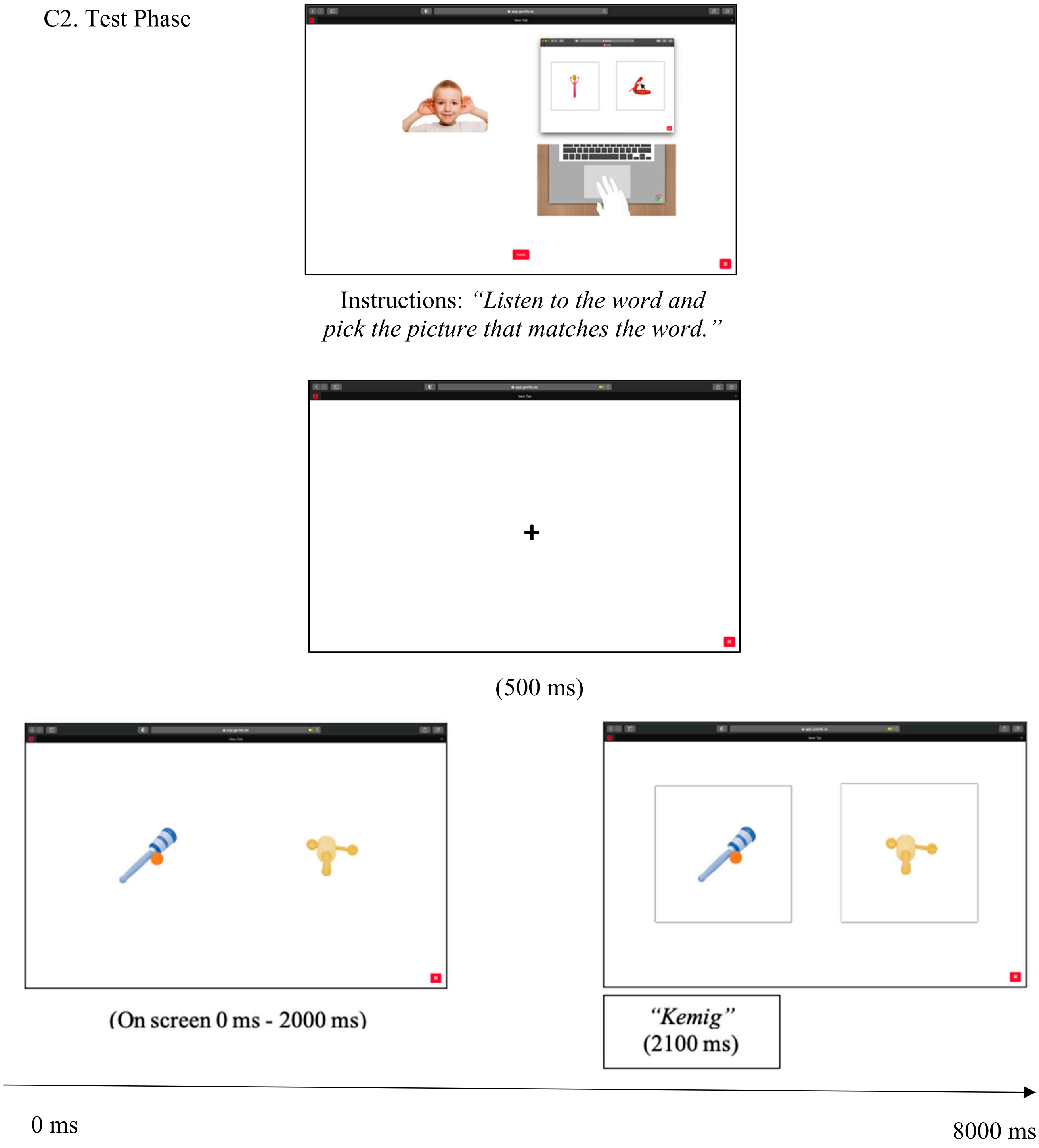

Note. Test instructions were only presented once at the beginning of the task. A 500 inter-stimulus-interval was presented before each test trial. In each test trial, the target word was produced once at 2100 ms, and response buttons immediately appeared around the novel objects. Participants had 4000ms after word onset to select a novel object. Each trial was approximately 8000 ms.
